# Cytotoxic Effects of Coated Gold Nanoparticles on PC12 Cancer Cell

**DOI:** 10.22086/gmj.v0i0.1110

**Published:** 2018-07-13

**Authors:** Hadi Zare Marzouni, Fazel Tarkhan, Amir Aidun, Kiana Shahzamani, Hamid Reza Jahan Tigh, Sareh malekshahian, Hamed Esmaeil Lashgarian

**Affiliations:** ^1^Department of Immunology, faculty of Medicine, Mashhad University of Medical Sciences, Mashhad, Iran; ^2^Student Research Committee, Lorestan University of Medical Sciences, Khorramabad, Iran; ^3^National Cell Bank of Iran, Pasteur Institute of Iran, Tehran, Iran; ^4^Tissues and Biomaterials Research Group (TBRG), Universal Scientific Education and Research Network (USERN), Tehran, Iran; ^5^Hepatitis research Center, Lorestan University of Medical Science , Khorramabad, Iran; ^6^Department of biotechnology, Islamic Azad University, Urmia branch, Urmia, Iran

**Keywords:** Gold Nanoparticle, Cancer, Apoptosis, ROS, LDH

## Abstract

**Background:**

The use of gold nanoparticles in medicine and especially in cancer treatment has been of interest to researchers. The effectiveness of this nanoparticle on cells significantly depends on the amount of its entry into the cells. This study was performed to compare the rate and mechanism of effect of gold nanoparticles coated with different amino acid on PC12 cancer cell line.

**Materials and Methods:**

The PC12 cells line were exposed to various concentrations of amino acid coated and uncoated gold nanoparticles (0.5, 2.5 and 5 μM). Cell death rate was determined according to level of Lactate dehydrogenase (LDH) release from cells and MTT assay. In addition cell morphology and the amount of Cellular Reactive oxygen species (ROS) were studied.

**Results:**

The uncoated gold nanoparticles have shown minor effects on cellular life. Gold nanoparticles coated by tryptophan at high concentrations (2.5, 5 and 25μM) increase in cancer cells metabolic activity. Gold nanoparticles coated by Aspartate also produce the largest amount of LDH and ROS in cancer cells and therefore caused of highest rate of apoptosis.

**Conclusion:**

The results showed that the nanoparticles coated with amino acids are affected on cellular metabolism and apoptosis more than uncoated nanoparticles. Also the smallest coated nanoparticles (coated by aspartate) have the most influence and by increasing the size, this effect was reduced.

## Introduction

At current, the cancer after cardiovascular disease is the second leading cause of death in the world [1]. To reduce the burden of these diseases people’s awareness and attitude and devise more effective drugs and new treatment methods is important [2]. There are different factors that involving appearance of a cancer cell in a normal tissue. All of them are leading to damage to cells’ DNA. Thus, damage to DNA (genetic material), is the most important cause to being cells cancerous. Genes are database of cells and control their activities. The damaged gene can cause large differences between cancer cells and normal cells, among which we can point out to a difference in the cytoplasm, nucleus and cell membrane [3].

These differences can be used to identify and specific destruction of cancer cells as well. PC12 cell line is a cancerous cell line that is isolated from rat’s pheochromocytoma cells and it had cultured in a specific medium. This cell line can show the characteristics of nervous cells [4, 5].

Surgery, chemotherapy and radiotherapy are three main methods of cancer treatment. Surgery is invasive treatment in which tumor and in some cases whole organ interested is removed. In chemotherapy, drug is used to destroy cells that have a lot of division power is used ionizing radiations, which leads to the damage to some normal cells as well. In radiotherapy, ionizing radiation is used to kill cancer cells that cause damage to healthy cells located in the radiation field [6].

Therefore, the radiotherapy dose increase practically is impossible [7] and to increase the efficiency of treatment, is used of the compounds to increase radiation resistance of healthy tissue and the radiosensitivity of tumor tissue [8]. In many cases, the mechanism of sensitizing substances effect is production of ROS free radicals. In relation to sensitizers, numerous studies have been done on different physical and chemical composition [9-11] that in the meantime, nanoparticles have special status that among these we can point out to the gold nanoparticles, carbon nanotubes and metal nanoparticles [12-19].

Nanomaterials with oxidative stress and lipid peroxidation play a key role in the damage to the DNA, membrane destruction and cell death which can lead to an effective treatment method through minimize the side effects of treatment, leading to the destruction of the tumor [20, 21].

Among these gold nanoparticles are of great importance. Mustafa Al-Saeed and his father, Ivan Al-Saeed research group studies show that gold nanoparticles coated with antibodies against the cancer cells are able to connect effectively to the cancerous cells.

Many cancer cells contain a protein called epidermal growth factor receptor (EFGR) on its surface that mainly is not found in healthy cells in the human body. These researchers binding gold nanoparticles to an antibody EGFR (with anti EFGR) have been able to attach mentioned nanoparticles to cancer cells and cause specific destruction of cancer cells with gold nanoparticles [22, 23].

Therefore, in this study the toxicity of gold nanoparticles uncovered and coated with amino acids valine and phenylalanine, tryptophan, glutamate and aspartate on the PC12 cells has been studied. Our main goal is to examine how we enter the more nanoparticles into cells and the idea of the study is that does amino acids help or not to better entering of particles into the cell.

## Materials and Methods

Cell culture medium, RPMI 1640 (Caisson Laboratories, Logan, UT, USA), fetal bovine serum (Caisson Laboratories, Logan, UT, USA), horse serum (Gibco Company, Grand Island, NY, USA), Solutions streptomycin (CMG Company, Isfahan, Iran), penicillin and Trypsin / EDTA (Invitrogen, Paisley, Scotland) , Cell culture plates (Nunc, Roskilde, Denmark), Tetrachloroauric acid (Sigma, MO, USA), L-aspartic acid (Sigma, MO, USA), L- glutamic acid (Sigma, MO, USA), L- tryptophan (Sigma, MO, USA), L- phenylalanine (Sigma, MO, USA), L- valine (Sigma, MO, USA), Trisodium citrate (Sigma, MO, USA), Trypan blue (Sigma, MO, USA), Sodium azide (Sigma, USA), Congo red (Sigma, MO, USA), 3-(4,5-Dimethylthiazol-2-yl)-2,5-diphenyltetrazolium bromide-MTT (Sigma, Germany), Acridine orange (AO) and Ethidium bromide (EtBr) (AB, Uppsala, Sweden), PC12 cell line from the Pasteur Institute of Iran, 2′,7′-Dichlorofluorescin diacetate from Molecular Probes (Eugene, OR, USA), Dimethyl sulfoxide (DMSO) (Merck, Darmstadt, Germany).

In this empirical study, the effect of five amino acids contains , phenylalanine-coated, tryptophan-coated, glutamate-coated, aspartate-coated and valine-coated gold nanoparticles on type and death rate of PC12 cancer cells was investigated.

### Cell Culture

In this study, PC12 cells obtained from the Pasteur Institute of Iran were used for in vitro culture. Cells were cultured in RPMI 1640 (Gibco) medium along with 0.2% (w/v) bovine serum albumin (BSA), 1% (v/v) L-glutamine and 1% (v/v) non-essential amino acids (NEAA) inside T-25 (NUNC) cell culture flasks, in an incubator containing 5% CO2 and adequate humidity at 37°C. After 48 h, the old culture medium was replaced with a new culture medium. Using trypsin–EDTA solution (200 unit/ml), cell passage was done when they occupied 70 to 80% of the flask [24-26].

### Production of Gold Nanoparticles without Coating

In order to prepare gold nanoparticles without cover, first (HAuCl_4_, 254169)was dissolved in water with 0.01M molar concentration and its ionic strength and pH were 0.005M and 7.8 respectively, which was set by the phosphate buffer system. A non-aqueous phase (toluene C_6_H_5_CH_3_) containing sodium tetraburohydrate of 0.2M concentration was also prepared separately. Then, both phases were added together and shaken. After excellent phase separation at 50°C and under low pressure using the rotary device, the solvent was removed. Finally, gold nanoparticles settled at the bottom of the flask and PBS with ionic strength and pH of 0.005M and 7.8, respectively were dispersed and a homogenized solution was obtained. The properties of the colloidal solution mentioned were clear and red. Next, the absorption spectrum of the gold nanoparticles prepared and also their frequency distribution were determined [27, 28].

### Production of Gold Nanoparticles Coated with Amino Acids

To prepare the coated nanoparticles, first, 300 ml amino acid solution of 25 µM concentration was added in a beaker containing 15 ml of deionized water. Then the beaker was placed on a heater until the solution reached boiling point, upon which 1 ml of 1 µM HAuCl_4_ solution was added. When the reaction was finished and the color changed, the container was immediately placed in an ice bath to cool the colloidal solution. Finally, the colloidal solution was filtered with a 0.2micron filter. This experiment was performed with different coatings of amino acids such as valine, phenylalanine, tryptophan, glutamate and aspartate [29, 30]. Scanning electron microscope (SEM) was used to evaluate the structure of amino acid-coated gold nanoparticle.

### Characterization of Nanoparticles

The size of the nanoparticles was measured by using dynamic light scatter (DLS, n = 3, Zetasizer Nano, Malvern Instrument Co., UK) and the formation of the nanoparticles was analyzed with a UV-vis spectrophotometer (Lambda 35, Perkin Elmer, USA).

Transmission Electron Microscope (TEM) micrographs were obtained on HITACHI HF-2200TU Transmission Electron Microscope operated at 200 kV as the accelerating voltage. The specimens were prepared on the copper grid coated with a carbon micro grid membrane and then dried.

### Studying the Effects of Different Gold Nanoparticle Samples on Cancer Cells

In order to study the possible effect of amino acid coating on toxicity of gold nanoparticle, PC12 cancer cells were exposed to various concentrations of gold nanoparticles (0.5, 2.5 and 5 μM). Cell death rate was assessed according to level of LDH (lactate dehydrogenase) release from cells. Activity rate of LDH enzyme in supernatant and lysed cellular sediment (in control group) was measured by spectrophotometry (LDH Cytotoxicity Assay Kit). Percentage of total LDH released into the culture medium was calculated by the following equation:

Percentage of total LDH released=

(LDH in culture medium)/(LDH in culture medium+LDH in lysed cellular solution) ×100

Simultaneously, MTT assay was done to determine cell viability. In addition, to examine cell morphology, control and treatment cells with different gold nanoparticle samples were studied by phase contrast microscopy. In order to study the form of cell death induced by phenylalanine-coated, tryptophan-coated, glutamate-coated, aspartate-coated, valine-coated and uncoated nanoparticles, Acridine Orange/Ethidium Bromide (AO/EB) staining was used. In this form of staining, type and rate of cell death is specified. Moreover, DNA in cells that undergo apoptotic death is dense and fragmented, whereas necrotic cells reveal dense and unsliced chromatin [31]. Finally, the amount of ROS within cell was measured using fluorescence probes 2’, 7’-dichlorofluorescin diacetate (DCFH-DA) [32].

### Statistical Analysis

The collected data were analyzed using SPSS 16 software, one-way analysis of variance (ANOVA) and Tukey’s test, while P<0.05 was considered significant.

## Result

**Figure-1:**
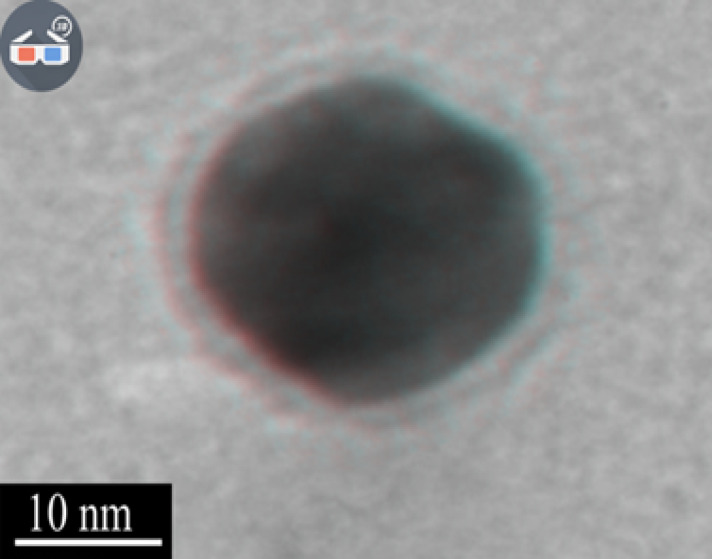
Scanned electron micrographs of the nanoparticles coated with phenylalanine amino acid (3D)

In order to Assessment of the structure of gold nanoparticles coated with amino acids electron microscope was used. SEM micrographs obtained show amino acid coatings in the form of a halo around nanoparticles (Figure-1). The mean diameter of gold nanoparticles in the case of uncoated and coated with amino acids are given in the Table-1.

**Table-1:** Diameter of Gold Nanoparticles Coated with Amino Acids

Nano-Particle	The Average Size of Nanoparticles (nm)
**No cover**	10 ± 0.2
**Coated with Aspartate**	16 ± 0.3
**Coated with Valine**	24 ± 0.4
**Coated with Phenylalanine**	27 ± 0.4
**Coated with Glutamate**	28 ± 0.5
**Coated with Tryptophan**	35 ± 1

Incubating PC12 cancer cells with coated gold nanoparticles show varying results. The uncoated gold nanoparticles have shown minor effects on cellular life and the degree of liberation of lactate dehydrogenase enzyme to the culture medium, when compared with control cells. At the maximum concentration, it has decreased the metabolic activity only by 14% and has increased the liberation degree of lactate dehydrogenase enzyme to the culture medium by 10%.

Incubation of PC12 cell line with gold nanoparticles coated with valine shows that these nanoparticles have a very small effect in low concentrations on cell viability, but at high concentrations in a dose-dependent manner could have a significant impact on have metabolic activity, so that the highest concentration of 25 µM, cell survival has been reduced compared to control cells, about 47 percent. Evaluation of the integrity of the membrane structure also shows this nanoparticle in the highest concentration has increased lactate dehydrogenase enzyme release into the culture medium, as compared to control cells.

Gold nanoparticles coated with phenyl alanine have had no effect on cellular life at low concentrations, with these effects being observable only at high concentrations, such that the degree of its effect on metabolic activity at the highest concentration reaches around 40%, when compared with control cells.

Gold nanoparticles coated with tryptophan not only have not been able to decrease the metabolic activity of cancer cells, but at high concentrations they have brought about around 20% increase in metabolic activity, when compared with control cells.

Gold nanoparticles coated with aspartate and glutamate also suggests decreased metabolic activity and increased membrane destruction of the cell, in comparison with control cells in a dose-dependent mode. The gradient of these changes has been greater with regard to gold nanoparticles with aspartate coating, such that at the concentrations of 0.5, 2.5, 5, 12.5, and 25 micromole on this nanoparticle, the cellular life has diminished by 13, 20, 23, 53, and 84% respectively when compared with control cells, while the degree of liberation of lactate dehydrogenase enzyme into the culture medium has shown 12, 17, 24, 40, and 74% increase when compared with control cells. However, gold nanoparticles with glutamate coating at the highest concentration have shown only 30% reduction in metabolic activity and 27% increases in the degree of liberation of lactate dehydrogenase enzyme into the culture medium of PC12 cancer cells, when compared with control cells.

**Figure-4:**
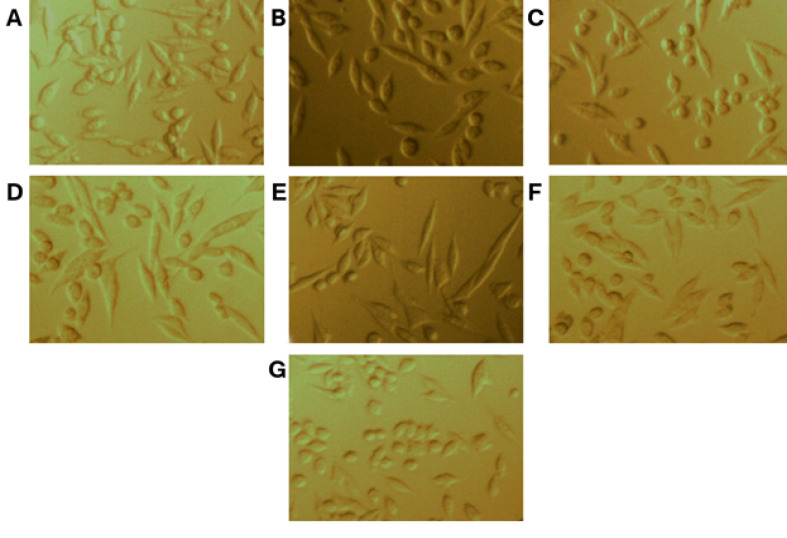
Phase contrast microscopy of PC12 cells treated with various types of gold nanoparticles (10X). **A**: control, **B**: incubated with uncoated nanoparticles, **C**: incubated with nanoparticles coated by valine, **D**: incubated with nanoparticles coated with phenylalanine, **E**: incubated with nanoparticles coated with tryptophan, **F**: incubated with nanoparticles coated with glutamate, **G**: incubated with nanoparticles coated with aspartate

Results showed that PC12 cancer cells usually have fusiform morphology. During incubation with uncoated gold nanoparticles, the morphological shape of cells changes from fusiform to spherical (Figure-4).

**Figure-5:**
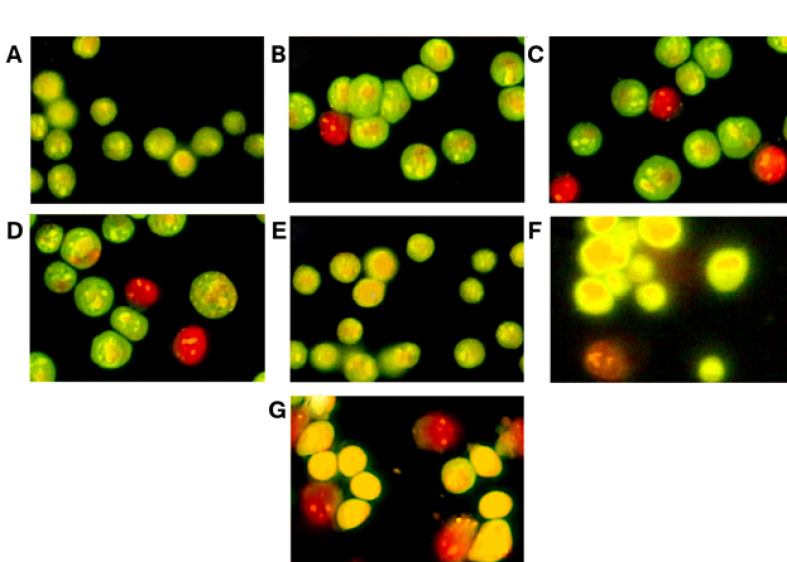
Apoptosis/necrosis induction by 25 μM gold nanoparticles (10X). **A**: Acridine Orange/Ethidium Bromide (AO/EB) staining in untreated cells, **B**: treated with uncoated nanoparticles and **C**: treated with valine-coated gold nanoparticles. **D**: treated with phenylalanine-coated gold nanoparticles. **E**: treated with tryptophan-coated gold nanoparticles. **F**: treated with glutamate-coated gold nanoparticles. **G**: treated with aspartate-coated gold nanoparticles.

In addition, the results demonstrated that much of cell death due to incubation with uncoated gold nanoparticles in Acridine Orange/Ethidium Bromide (AO/EB) staining is in the form of apoptosis (Figure-5).

**Figure-6:**
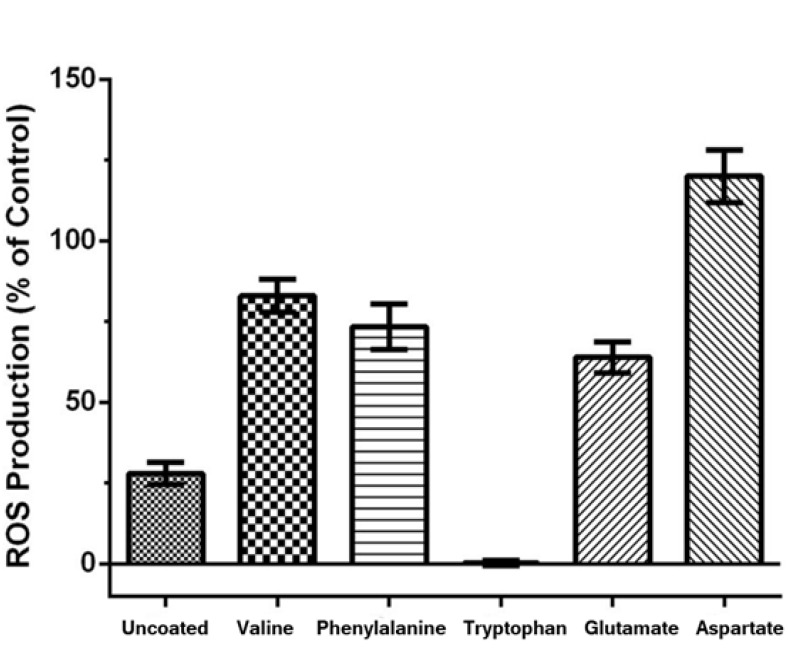
The level of ROS in PC12 cancer cells treated with different types of gold nanoparticles with concentration of 25 µM **A**: incubated with uncoated nanoparticles, **B**: incubated with nanoparticles coated with valine, **C**: phenylalanine, **D**: tryptophan, **E**: Glutamate, **F**: Aspartate

Results also imply that proximity of PC12 cancer cells to 25 μM gold nanoparticles coated with valine, phenylalanine, Glutamate, Aspartate for 48 hours increased ROS production rate within cells, compared with control cells (P>0.001) (Figure-6).

On the contrary, Proximity of PC12 cancer cells to tryptophan for 48 hours increased ROS production rate within cells in compared with control cells. Figure-6 show the level of ROS in PC12 class cancer cells treated with different types of gold nanoparticles with concentration of 25 µM.

As can be seen, the most increase in cell death belongs to gold nanoparticles coated with Aspartate. Difference in cell death rate between the group treated with uncoated gold nanoparticles and gold nanoparticles coated with Aspartate was statistically significant (P<0.001).

## Discussion

**Figure-2:**
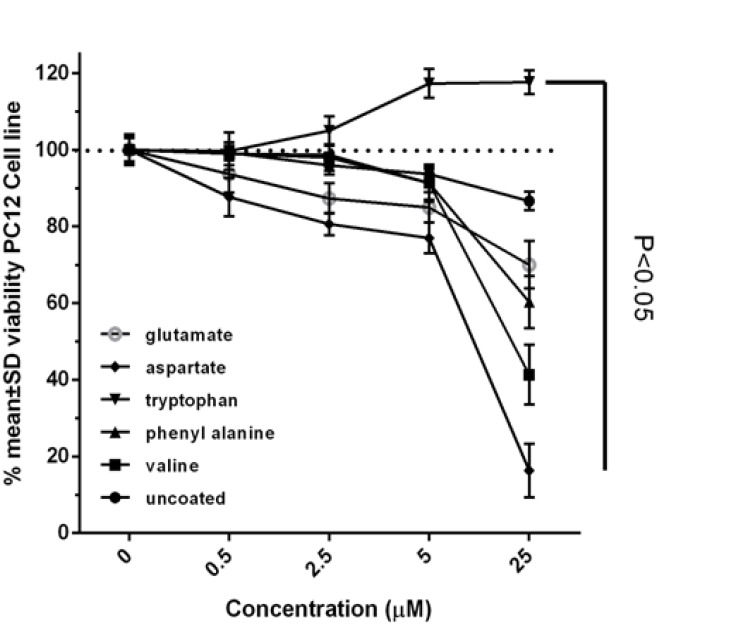
Cell viability (MTT), after 48 h of PC12 cell line incubation with gold nanoparticles coated and uncoated

**Figure-3:**
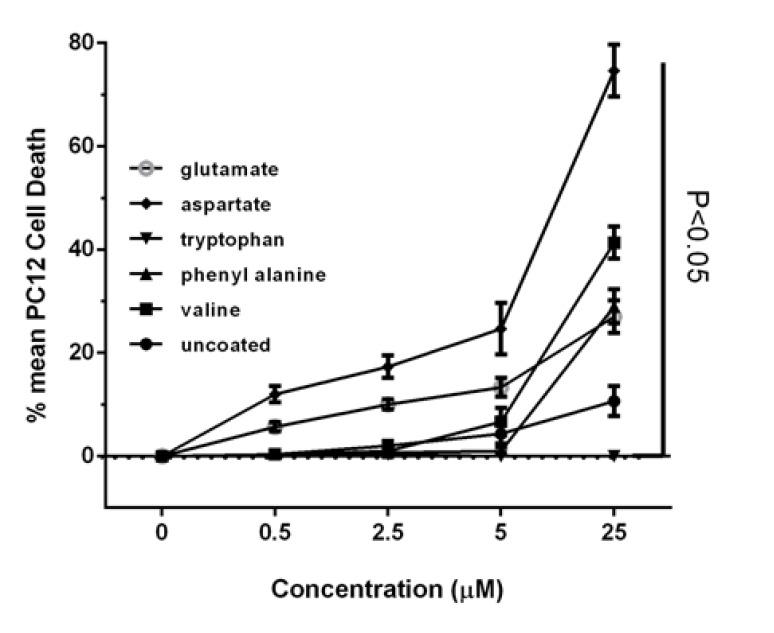
Percent of PC12 Cell Death after 48 h incubation with coated gold nanoparticles and uncoated nanoparticle

In this study, the preparation of gold nanoparticles coated by chemical reduction method with the use of different amino acids was done. To evaluate the cytotoxicity of the nanoparticles various techniques, including measuring intracellular ROS, lactate dehydrogenase activity assay, and MTT assay were used. The study demonstrated that the coating of gold nanoparticles with valine, phenylalanine, glutamate and aspartate increased cell death rate among PC12 cancer cells [33], compared with uncoated gold nanoparticles. On the other hand, gold nanoparticles coated with tryptophan, the largest amino acid, lead to reduced cytotoxicity of the gold nanoparticles. Some studies have shown that the use of gold nanoparticles for the treatment of cancer cells does not have any adverse effect on normal cells and vital organs [34, 35]. For years, researchers have used PC12 cells to study cancer cells and biological issues [36]. In this study, differences in shape, size and surface charge of gold nanoparticles increased its toxic effects on cancer cells [37-39]. Our studies on the integrity of membrane covering showed that the presence of amino acids can cause disruption of membrane integrity by increasing interactions between nanoparticles and cell surface or by creating pores in the plasma membrane [40, 41]. The results show that the disruption of membrane integrity is approximately dependent on the size of gold nanoparticles coated with amino acids, cell viability and the cytoplasmic release of lactate dehydrogenase enzyme. As can be seen in Table-1, the smallest gold nanoparticle is coated with aspartate, which is the most cytotoxic nanoparticle (Figure-2) and has the greatest impact on the induction of apoptotic death in cancer cells; thus, indicating a strong interaction of this nanoparticle with death receptors on the surface of cancer cells. An increase of nearly 50% in lactate dehydrogenase in the medium treated with aspartate-coated nanoparticles shows that it is better absorbed in comparison with non-coated nanoparticles due to better contact with cell receptors and because of its smaller size (Figure-3). Also, the results of this survey show that the level of ROS production by cells adjacent to the coated nanoparticles was considerably more than that of the uncoated nanoparticle (P≤0.05). It indicates apoptotic death induction in these cells by gold nanoparticles through increased production of active oxygen species (ROS). ROS plays a major role in redox mechanisms in apoptotic signaling and control. Evidences to date have shown that redox-dependent mechanisms play a central role in mitochondria-to-cytosol release of cytochrome c and apoptosis initiation. In Pan and colleagues’ study, gold nanoparticles with 1.4 nm diameter, coated with triphenylphosphinemonosulfonate (TPPMS) killed cells by necrosis; mitochondrial permeability transition was the main cause of cell death. When permeability transition is induced and mitochondrial activity quickly depletes ATP supply, cells die by necrosis. Then, the integrity of the plasma membrane is disrupted. The measurement of caspase 3/7 activity also revealed that necrosis not apoptosis occurred in these cells [42].

Cytotoxicity is reduced by increasing the size of nanoparticles. Cell death by the mechanisms of necrosis and apoptosis (death planned) occurs with different histological and biochemical characteristics. In necrosis, the death driver (such as local anemia) is often the direct cause of cell death. On the other hand, in apoptosis, death stimulation enables a cascade of coordinated events to destroy the cells. Sometimes, markers of apoptosis and necrosis are seen simultaneously in one cell therefore it is indicative of more than one type of cell death in the cell [43-45]. In order to study apoptosis induced by different types of gold nanoparticles, ethidium bromide staining / acridine orange was used. Cells exposed for 48h in the presence of different types of gold nanoparticles (with 25 µM concentration), are often seen as red with bright spots (indicating the existence of fragmented DNA) showing induction of apoptotic death.It has been reported that DNA in cells undergoing apoptotic death becomes as dense and fragmented, while necrotic cells show condensed chromatin and fragmentation [46]. Several studies have shownthat gold nanoparticle alone has no cell cytotoxicity [47-49]. In general, the induction of programmed cell death is one of the attractive applicationsof nanotechnology. The cell death pathway can include the activation of pro-apoptotic events of mitochondria beginning with the release of cytochrome C [42, 50].

## Conclusion

The results showed that coated gold nanoparticles effects on cell death by apoptosis process which it can kill cancer cells. Aspartate amino acid showed best performance to eliminate PC12 cancer cells.

## Conﬂicts of Interest

The author(s) indicated no potential conﬂicts of interest.
